# Association Between Antihypertensive Medication Use and Breast Cancer: A Systematic Review and Meta-Analysis

**DOI:** 10.3389/fphar.2021.609901

**Published:** 2021-05-13

**Authors:** Yuxiu Xie, Men Wang, Peng Xu, Yujiao Deng, Yi Zheng, Si Yang, Ying Wu, Zhen Zhai, Dai Zhang, Na Li, Nan Wang, Jing Cheng, Zhijun Dai

**Affiliations:** ^1^ Department of Breast Surgery, The First Affiliated Hospital, College of Medicine, Zhejiang University, Hangzhou, China; ^2^ Cancer Center, Union Hospital, Tongji Medical College, Huazhong University of Science and Technology, Wuhan, China; ^3^ Department of Oncology, The Second Affiliated Hospital of Xi’an Jiaotong University, Xi’an, China

**Keywords:** antihypertensive drugs, breast cancer, meta-analysis, prognosis, risk

## Abstract

**Background:** The prevalence rate of hypertension and breast cancer increases with advancing age. Renin-angiotensin system inhibitors (RASIs), β-blockers (BBs), calcium channel blockers (CCBs), and diuretics are widely used to treat patients with hypertension. Although, the association between the use of antihypertensive medication and breast cancer has been highly debated, recent evidence supporting this association remains controversial.

**Objective:** To evaluate the association between the use of antihypertensive medication and the risk of breast cancer and its prognosis.

**Methods:** This study was conducted using data from the PubMed, Embase, and Cochrane Library databases retrieved for the period from January 2000 to April 2021. Articles and their references were checked and summary effects were calculated using random- and fixed-effects models. Heterogeneity test and sensitivity analysis were also performed.

**Results:** This meta-analysis included 57 articles, which were all related to breast cancer risk or prognosis. Assessment of breast cancer risk using the pooled data showed that the use of BBs or CCBs or diuretics was associated with increased cancer risk [BB: relative risk (RR) = 1.20, 95% confidence interval (CI) = 1.09–1.32; CCBs: RR = 1.06, 95% CI 1.03–1.08; diuretics: RR = 1.06, 95% CI 1.01–1.11]. Long-term use of diuretic increased the risk of breast cancer (RR = 1.10, 95% CI 1.01–1.20), whereas long-term RASIs treatment reduced the risk (RR = 0.78, 95% CI 0.68–0.91). In addition, we found that diuretic users may be related to elevated breast cancer-specific mortality [hazard ratio (HR) = 1.18, 95% CI 1.04–1.33], whereas using other antihypertensive medications was not associated with this prognosis in patients with breast cancer.

**Conclusion:** Using CCBs, BBs, and diuretics increased the risk of breast cancer. In addition, diuretics may elevate the risk of breast cancer-specific mortality. The long-term use of RASIs was associated with a significantly lower breast cancer risk, compared with non-users. Thus, this analysis provides evidence to support the benefits of the routine use of RASIs in patients with hypertension, which has important public health implications.

## Introduction

In 2017, hypertension was identified as a major risk factor for cardiovascular disease globally, accounting for 10.4 (9.39–11.5) million deaths and 218 (198–237) million disability-adjusted life-years (Stanaway et al., 2018). Fortunately, antihypertensive therapy significantly reduces the risk of cardiovascular disease and death in various populations. A meta-analysis showed that reducing the systolic blood pressure by 10 mm Hg would reduce the risk of major cardiovascular disease, coronary heart disease, stroke, and heart failure by 20, 17, 27, and 28%, respectively, whereas all-cause mortality was reduced by 13% (Ettehad et al., 2016). As an effective measure to control blood pressure, antihypertensive medications are commonly prescribed worldwide, and many patients take these drugs for a long period.

Several studies have found that breast cancer is associated with a variety of antihypertensive medications, including angiotensin-converting enzyme inhibitors/angiotensin receptor blockers (ACEIs/ARBs), β-blockers (BBs), calcium channel blockers (CCBs), and diuretics. However, these results remain controversial. Raebel et al. (2017) concluded that long-term use of CCBs was not related to breast cancer risk and long-term use of ACEIs might protect against breast cancer. The Chang et al. (2016) conducted a nested case-control study of 794,533 women and did not find any relationship between antihypertensive medication use and breast cancer risk. In contrast, other studies found that long-term treatment with CCBs was associated with a higher risk of breast cancer; however, no association was observed with the use of diuretics, BB, and renin-angiotensin system inhibitors (RASIs) (Li et al., 2013; Gomez-Acebo et al., 2016). A population-based case-control study demonstrated that treatment with ACEIs for more than 5 years increased breast cancer risk by 14% [odds ratio (OR) = 1.14, 95% confidence interval (CI) = 1.06–1.22] (Hallas et al., 2012).

A recent systematic review of observational studies concluded that BB reduced the risk of breast cancer recurrence (Caparica et al., 2021), whereas ACEIs and CCBs were not associated with breast cancer development (Zhao et al., 2017a; Naghibzadeh et al., 2020). In addition, the result of a meta-analysis suggested an association between CCBs use and breast cancer risk (Wright et al., 2017). However, uncertainty still exists regarding the effect of long-term use of antihypertensive medications and a potential association between different populations, breast cancer sub-types, or both.

This question remains controversial and, therefore, in this updated meta-analysis, we aimed to assess the association between the use of various classes of antihypertensive medication and breast cancer risk, prognosis [breast cancer-specific mortality, recurrence, overall survival (OS), and disease specific survival (DSS)].

## Materials and Methods

### Data Sources and Search Strategy

Supplementary Table S1 shows the strategy we used to conduct a comprehensive literature search of the PubMed, Embase, and Cochrane Library electronic databases without any restriction regarding geographical parameters and publication type or language. We reviewed research articles published over a span of nearly 20 years rather than those published in the 1990s, because we considered that the technique and treatment strategies that affect patient prognosis and survival have improved with time. The search was based on the framework of adult populations exposed to antihypertensive medication compared with non-users and the articles were evaluated for diagnosis or progress of breast cancer. Based on a combination of MeSH terms, keywords, and substance names, we conducted the following string search: “antihypertensive medication,” “calcium channel blockers,” “beta blockers,” “angiotensin-converting enzyme inhibitors,” “angiotensin receptor blockers,” “renin-angiotensin system inhibitors,” or “diuretics” combined with “breast cancer” or “breast carcinoma.” In addition, the reference lists of other reviews or meta-analyses were manually searched to identify additional related articles. This meta-analysis is not registered in the PROSPERO database, but the process is based on the Preferred Reporting Items for Systematic Reviews and Meta-Analyses (PRISMA) guidelines (Page et al., 2021). The PRISMA 2020 checklist was shown in Supplementary Table S2.

### Selection Criteria

Studies fulfilling the following inclusion criteria were included: 1) the exposed group included individuals who had received antihypertensive medication; 2) the study had a comparative design: antihypertensive medication users vs. non-users; 3) the outcome was breast cancer risk, breast cancer-specific mortality, recurrence, or survival (DSS or OS); 4) the study reported the relative risk (RR), OR, or hazard ratio (HR) with corresponding 95% CIs or sufficient data to calculate these parameters. When the results of multiple studies were from the same database, the study including the largest number of participants was included. The following types of studies were excluded from this meta-analysis: meta-analyses, systematic or narrative reviews, case studies, experimental laboratory articles, conference abstracts, commentaries, randomized controlled trials, repeated publications, or if the reference group was administered another class of antihypertensive medication.

### Data Extraction and Quality Assessment

Two of the authors (Yuxiu Xie and Men Wang) extracted data from the included studies and any disagreements between them were resolved by discussions with between the two other relevant co-authors. We extracted the following information from the selected articles: first author and year of publication, source of data, country, study design, duration of follow-up, study outcome, sample size of participants, and covariates used for the adjustment of confounders. The risk estimate adjusted for the largest number of confounding factors was extracted.

The nine-star Newcastle–Ottawa Scale (NOS) was used to assess the methodological quality of cohort studies and case-control studies (Stang, 2010; Ma et al., 2020). A score of ≥7 was considered to indicate high quality.

### Statistical Analysis

We conducted this meta-analysis to assess the association between the use of antihypertensive medication and breast cancer risk and prognosis. The summary risk estimates are presented as forest plots. The *I* ^2^ test was used to assess potential heterogeneity between individual studies, and an *I* ^2^ > 50% indicated significant heterogeneity (Higgins et al., 2003). A fixed-effects model was used when there was no significant heterogeneity among the studies; whereas, the random-effects models was used when there was. To explore the source of heterogeneity and evaluate the potential impact of variables, we conducted subgroup analyses based on study design, country, and duration of antihypertensive medication use in years (e.g., <5, 5–10, ≥10 years). Articles with results that showed separate risk estimates based on different classes of CCBs or a different pathological pattern of breast cancer without a summary result were treated as different studies according to the result. Funnel plots were generated and examined visually, and gauged Egger’s tests were performed to assess publication bias (Egger et al., 1997). Sensitivity analyses were conducted excluding one study at a time to test the robustness of this association.

## Results

### Characteristics and Quality of Studies

**FIGURE 1:**
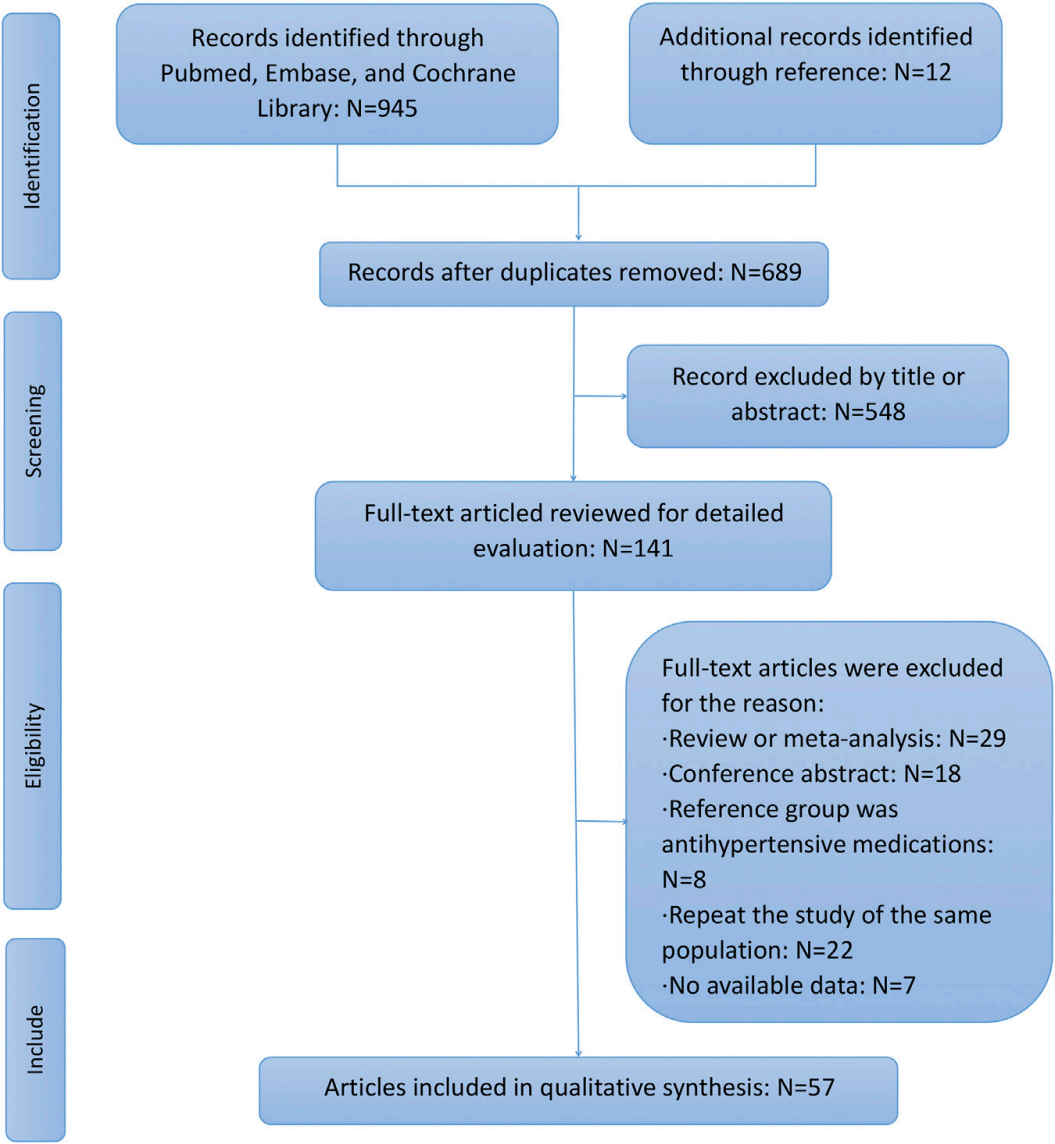
Flow chart of the study procedure.

A total of 842 potentially eligible studies were retrieved from the selected databases during the initial search. After removing duplicates and further screening the titles and abstracts, 26 studies on breast cancer risk (Meier et al., 2000; Li et al., 2003; Gonzalez-Perez et al., 2004; Fryzek et al., 2006; Largent et al., 2006; Davis and Mirick, 2007; Van Der Knaap et al., 2008; Coogan et al., 2009; Largent et al., 2010; Huang et al., 2011; Hallas et al., 2012; Lee et al., 2012; Mackenzie et al., 2012; Biggar et al., 2013; Li et al., 2013; Saltzman et al., 2013; Devore et al., 2015; Numbere et al., 2015; Azoulay et al., 2016; Chang et al., 2016; Gomez-Acebo et al., 2016; Wilson et al., 2016; Brasky et al., 2017; Raebel et al., 2017; Busby et al., 2018b; Zheng et al., 2021), and 30 studies on breast cancer prognosis (Powe et al., 2010; Barron et al., 2011; Ganz et al., 2011; Melhem-Bertrandt et al., 2011; Shah et al., 2011; Şendur et al., 2012; Holmes et al., 2013a; Holmes et al., 2013b; Botteri et al., 2013; Cardwell et al., 2013; Chae et al., 2013; Sorensen et al., 2013; Boudreau et al., 2014; Cardwell et al., 2014; Sakellakis et al., 2014; Babacan et al., 2015; Chen et al., 2015; Springate et al., 2015; Cardwell et al., 2016; Choy et al., 2016; Chen et al., 2017; Spera et al., 2017; Busby et al., 2018a; Musselman et al., 2018; Cui et al., 2019; Takada et al., 2019; Modi et al., 2020; Santala et al., 2020; Shih et al., 2020; Wei et al., 2020) and one study on both risk and prognosis (Sorensen et al., 2000) finally met the inclusion criteria of this meta-analysis. The study selection process is depicted in a flow chart (Figure 1) and the characteristics of the included studies are summarized in Table 1. The sample sizes of the studies included for breast cancer risk ranged from 654 to 2,300,000 with a total of 3,726,281 participants, and that for breast cancer prognosis varied from 218 to 73,170 with a total of 270,745 participants. Although the adjusted covariates of individual studies differed, most risk estimates were adjusted for age, body mass index (BMI), alcohol intake, and hormone replacement therapy use. The quality scores in this analysis ranged from 6 to 8 stars (Supplementary Table S3).

**TABLE 1:** Characteristics of the articles included in the meta-analysis.

Author, Year	Country	Class of medication vs. reference group	Study period	Source of data	Participants	Design type	Outcomes	Adjustment/match for covariates	NOS
Azoulay et al. (2016)	United Kingdom	CCB vs. non	1995–2010	The United Kingdom General Practice Research Database	27,315	Cohort study	Risk	Age, year of cohort entry, excessive alcohol use, smoking status, body mass index, previous cancer, oophorectomy, use of antidiabetic drugs, aspirin, other nSaiDs, statins, hormone replacement therapy, oral contraceptives, use of angiotensin receptor blockers, angiotensin-converting enzyme inhibitors, beta-blockers, diuretics, and other antihypertensive drugs	7
Biggar et al. (2013)	Denmark	Diuretics vs. non	1995–2010	The Danish Register of Medicinal Products Statistics	2300000	Cohort study	Risk	Age and calendar-year	6
Brasky et al. (2017)	United States	CCB vs. non	1993–2010	Women’s Health Initiative	28,561	Case-control	Risk	Age, WHI-CT intervention assignment, education, race, BMI, physical activity, smoking, alcohol, and breast cancer screening	7
Busby et al. (2018a)	United Kingdom	CCB (verapamil) vs. non	1995–2010	The United Kingdom Clinical Practice Research Datalink	90,294	Case-control	Risk	Comorbidities (AIDS, cerebrovascular disease, chronic pulmonary disease, congestive heart disease, dementia, diabetes, diabetes with complications, ductal carcinoma *in situ*, hemiplegia, mild liver disease, moderate liver disease, myocardial infarction, peptic ulcer disease, peripheral vascular disease, renal disease, and rheumatological disease), confounder medications (aspirin, digoxin, hormone replacement therapy, metformin, oral contraceptive, and statin), deprivation, smoking status, alcohol consumption, and obesity, age, GP practice, and year of diagnosis	7
Chang et al. (2016)	China	BB, CCB, RASI vs. non	2001–2011	Taiwan National Health Insurance claims database	46,985	nested Case-control	Risk	socioeconomic status, diabetes mellitus, ischemic heart disease, myocardial infarction, heart failure, cerebrovascular disease, chronic kidney disease, chronic liver disease, chronic lung disease, depression, Charlson’s index, diuretics, human insulin, statin, fibrates, aspirin, hormone replacement therapy, number of lipid measurements, number of mammography, number of outpatient visits, number of hospitalizations, and length of hospital admission more than 7 days matched on age (within 5 years) and follow-up duration	8
Coogan et al. (2009)	United States	Diuretics vs. non	1976–2007	Boston, New York, Philadelphia and Baltimore	11,493	Case-control	Risk	Race (white, non-white), years of education, menopausal status, parity, body mass index, use of female hormones, use of oral contraceptives and alcohol use	8
Davis and Mirick (2007)	United States	BB, CCB vs. non	2000–2001	The Seattle metropolitan area	1247	Case-control	Risk	Parity, age at first pregnancy, mother/sister breast cancer, early double oophorectomy, oral contraceptive use, ever upper gastro-intestinal series, and ever smoker (all subjects); mother/sister breast cancer younger than age 45 and alcohol intake; hormone replacement therapy. 5-year age	6
Devore et al. (2015)	United States	BB, CCB, ACEI vs. non	1988–2012	The Nurses’ Health Study	148668	Cohort study	Risk	Age and body-mass index, height, oral contraceptive use, menopausal status, age at menopause, postmenopausal hormone use, parity and age at first birth, age at menarche, family history of breast cancer, history of benign breast disease, alcohol intake, physical activity level, smoking	8
Fryzek et al. (2006)	Denmark	BB, CCB, ACEI, ARB, Diuretics vs. non	1990–2002	The Central Population Register, The Danish Cancer Registry, The Pharmaco-Epidemiologic Prescription Database of North Jutland	20,088	Cohort study	Risk	Age, calendar period, HRT use, NSAID use, parity, and age at first birth	7
Gomez-Acebo et al. (2016)	Spain	BB, CCB, ACEI, ARB, Diuretics vs. non	2008–2013	The Multi Case-Control-Spain Study	3631	Case-control	Risk	Age, area of resident, education, body mass index, active smoking, alcohol intake, family history of breast cancer, age of menarche, age first full-term births, number of full-term births, menopausal status, hormonal therapy	7
Gonzalez-Perez et al. (2004)	United Kingdom	BB, CCB, ACEI, Diuretics vs. non	1995–2001	The United Kingdom General Practice Research Database	23,708	Nested case-control	Risk	Age, calendar year, BMI, alcohol intake, smoking status, HRT use, prior breast lump and/or biopsy, hypertension and all the variables in the table using logistic regression	8
Hallas et al. (2012)	Denmark	ACEI, ARB vs. non	2000–2005	The Danish Cancer Registry, the Danish National Registry of Patients, the Prescription Database of the Danish Medicines Agency and the Danish Person Registry	97,573	Case-control	Risk	A prior discharge diagnosis of chronic obstructive pulmonary disease as a crude marker of heavy smoking, a prior discharge diagnosis of inflammatory bowel disease, a modified Charlson Index that contains 19 categories of co-morbidity, match age and gender	8
Huang et al. (2011)	China	ARB vs. non	1998–2007	The Taiwan National Health Insurance database	109344	Cohort study	Risk	Age, gender, co-morbidities, and medications for hypertension control	7
Largent et al. (2010)	United States	CCB, ACEI, Diuretics vs. non	1995–2006	The California Teachers Study cohort	118700	Cohort study	Risk	Race, family history of breast cancer, age at first full-term pregnancy and number of full-term pregnancies combined variable, hormone therapy and menopausal status combined variable, lifetime physical activity, diabetes, body mass index, smoking history, alcohol use, hysterectomy, breastfeeding, and quartiles of percent calories from fat	7
Largent et al. (2006)	United States	Diuretics vs. non	1994–1999	The Cancer Surveillance Program of Orange County	654	Case-control	Risk	Age, body mass index (BMI), diabetes, smoking, alcohol use, menopausal status, family history of breast or ovarian cancer, age at first full-term pregnancy and education	6
Lee et al. (2012)	China	ACEI vs. non	2002–2008	The Taiwan National health Insurance Research Database	67,388	Case-control	Risk	Urbanization, income, diabetes mellitus, metformin usage, statin usage, estrogen usage, and progesterone usage	7
Li et al. (2003)	United States	BB, CCB, RASI, Diuretics vs. non	1997–1999	The westeren Washington State	1982	Case-control	Risk	Age	7
Li et al. (2013)	United States	BB, CCB, ACEI, ARB, Diuretics vs. non	2000–2008	The three-county Seattle-Puget Sound metropolitan area	2763	Case-control	Risk	Age, reference year, county, race/ethnicity, and recency of alcohol use	8
Mackenzie et al. (2012)	United Kingdom	Spironolactone vs. non	1987–2010	The United Kingdom General Practice Research Database	85,452	Cohort study	Risk	Age, calendar year of entry to study, Townsend score, use of combined oral contraceptive pill or hormone replacement therapy, history of benign breast disease, alcohol intake, body mass index, family history of breast cancer, use of drugs that may protect against breast cancer (aspirin, metformin), use of drugs causing gynecomastia (digoxin, finasteride, cimetidine, nifedipine), and history of hypertension, heart failure, or diabetes mellitus, socioeconomic score	7
Meier et al. (2000)	United Kingdom	BB, CCB, ACEI vs. non	1992–1997	The United Kingdom General Practice Research Database	17,861	Case-control	Risk	Smoking and body mass index, match age (same year of birth), physician practice, calendar date, and number of years of medical history	7
Numbere et al. (2015)	United Kingdom	BB, CCB vs. non	1987–2012	The Clinical Practice Research Datalink	320251	Case-control	Risk	Age, sex, smoking, alcohol use, and a number of potentially confounding comorbidities and coprescriptions	7
Raebel et al. (2017)	United States	CCB, ACEI vs. non	1997–2012	The Kaiser Permanente health-care system	111882	Cohort study	Risk	Age, body mass index, hysterectomy, diabetes, alcohol abuse, estrogen replacement, use of statins, mammography, and use of angiotensin receptor blockers, β-blockers, and/or diuretics, Kaiser Permanente site, race/ethnicity, education, and year of cohort entry, match Length of follow-up	6
Saltzman et al. (2013)	United States	BB, CCB, ACEI, Diuretics vs. non	1989–2001	Cardiovascular health study	3389	Cohort study	Risk	Age, alcohol use, income, age at menopause, waist-hip ratio	7
Van Der Knaap et al. (2008)	Netherlands	RASI vs. non	1991–2004	The Rotterdam Study	7821	Cohort study	Risk	Age, BMI, total pack-years, age at menarche and menopause, use of hormone-replacement therapy, number of children, diabetes mellitus, NSAID use, physical activity, hypertension, and myocardial infarction	7
Wilson et al. (2016)	United States	CCB vs. non	2003–2013	The Sister Study	17,782	Cohort study	Risk	Race/ethnicity, categorized body mass index, parity, age at menarche, menopause status, statin use, smoking status, hormone therapy use, and reported hours of physical activity per week	8
Zheng, G et al. (2021)	Sweden	BB (propranolol, metoprolol, atenolol and bisoprolol) vs. non	2005–2014	The Swedish Prescribed Drug Registry, The Swedish Cancer Registry	38,282	Cohort study	Risk	For propranolol was use of lipid-modifying agents, for metoprolol were family history of breast cancer, education level, income and use of cardiac therapy, antihypertensive, diuretics, calcium channel blockers, hormone replacement therapy and aspirin, for atenolol were family history of breast cancer, education level and use of antihypertensive, and for bisoprolol were use of cardiac therapy, diuretics, lipid-modifying agents and hormone replacement therapy, and personal history of chronic pulmonary disease	7
Babacan et al. (2015)	Turkey	RASI vs. non	2005–2012	Hacettepe Cancer Institute	218	Cohort study	OS, DFS	NA	7
Barron et al. (2011)	Ireland	BB (Propranolol, Atenolol) vs. non	2001–2006	The National Cancer Registry Ireland	5801	Case-control	BCSM	age, stage, grade, and comorbidity	8
Botteri et al. (2013)	Italy	BB vs. non	1997–2008	The European Institute of Oncology in Milan	800	Cohort study	BCSM, recurrence	Age, tumor stage, and treatment, peritumoral vascular invasion and use of other antihypertensive drugs, anti-thrombotics, and statins	7
Boudreau et al. (2014)	United States	ACEI, BB, CCB, Diuretics vs. non	1990–2008	The western Washington Cancer Surveillance System	4216	Cohort study	recurrence	All other medication classes of interest, age; diagnosis year; AJCC stage; hormone receptor status; primary treatment for initial breast cancer; endocrine therapy for the incident breast cancer); BMI; smoking; menopausal statu; Charlson co-morbidity score; diabetes; prescription non-steroidal anti-inflammatory medication use, Cox-2 inhibitors, and aspiri; and receipt of screening mammogram in the 12 months prior to events	8
Busby et al. (2018b)	United Kingdom	CCB vs. non	1998–2012	United Kingdom the Clinical Practice Research Datalink, and to death records from the Office for National Statistics	23,669	Cohort study	BCSM	Age at diagnosis, year of diagnosis, deprivation quintile, comorbidities, prior use of HRT or oral contraceptives, and treatment within six months of diagnosis	7
Cardwell et al. (2013)	United Kingdom	BB vs. non	1998–2007	United Kingdom clinical practice research datalink cohort	7132	nested Case-control	BCSM	Surgery within 6 months of diagnosis, chemotherapy within 6 months, radiotherapy within 6 months, tamoxifen, aromatase inhibitors, NSAID use, ACEI use, ARB use, statin use, hormone replacement therapy, comorbidities (pre-diagnosis, including myocardial infarction, cerebrovascular disease, congestive heart disease, chronic pulmonary disease, peripheral vascular disease, peptic ulcer disease and diabetes), and smoking, stage, restricted to individuals with an available stage record. match year and age at diagnosis	8
Cardwell et al. (2014)	United Kingdom	ARB, ACEI vs. non	1998–2006	United Kingdom clinical practice research datalink cohort	8541	Nested case-control	BCSM	Surgery, chemotherapy, radiotherapy, low dose aspirin, statins, comorbidities (myocardial infarction, cerebrovascular disease, congestive heart disease, chronic pulmonary disease, peripheral vascular disease, renal disease, peptic ulcer disease and diabetes), and smoking, tamoxife, aromatase inhibitors, hormone replacement therapy	8
Cardwell et al. (2016)	European	BB vs. non	1998–2012	The European Cancer Pharmacoepidemiology Network	55,252	Cohort study	BCSM	Age, year, stage and confounders	7
Chae et al. (2013)	United States	ACEI, ARB, BB vs. non	1995–2007	The MD Anderson Cancer Center	1449	Case-control	DSS, OS	Age, stage of disease, tumor grade, tumor subtype, LVI, and BMI	7
Chen et al. (2017)	United States	ACEI, ARB, BB, CCB, Diuretics vs. non	2007–2011	The linked Surveillance, Epidemiology and End-Results-Medicare database	14,766	Cohort study	BCSM, recurrence	Age at diagnosis, year of diagnosis, cancer stage, estrogen receptor status, receipt of complete first course treatment, receipt of chemotherapy, use of adjuvant hormone therapy, baseline diabetes, baseline hypertension at breast cancer diagnosis and use of other classes of antihypertensive medications	7
Chen et al. (2015)	United States	ACEI, BB, CCB, Diuretics vs. non	1990–2005	The Cancer Surveillance System	1010	Nested case-control	recurrence	adjuvant hormone therapy, radiation therapy and chemotherapy, match age, year of diagnosis, county, race/ethnicity, and cancer stage	7
Choy et al. (2016)	United States	BB vs. non	2000–2010	The City of Hope Cancer Center	1029	Cohort study	Recurrence	NA	6
Cui et al. (2019)	China	ACEI, ARB, BB, CCB, Diuretics vs. non	1996–2000	The Shanghai Women’s Health Study	2891	prospective study	OS, DSS	Age at diagnosis, sex, education, annual family income, body mass index, alcohol consumption, cigarette smoking status, Charlson Comorbidity Index score, year of cancer diagnosis, cancer stage, and cancer treatment. In model 2, we added a 6-month lag period into defining exposure in order to address potential reverse causation	7
Ganz et al. (2011)	United States	ACEI, BB vs. non	1997–2010	The LACE Study cohort	1779	Cohort study	BCSM, recurrence,OS	Age at diagnosis, race, stage of disease, pre-diagnosis BMI, adjuvant treatment, hormone receptor status, tamoxifen use, and self-reported hypertension and diabetes	7
Holmes et al. (2013a)	United States	ACEI, BB vs. non	1988–2008	The Nurses’ Health Study	4661	Cohort study	BCSM	Calendar year, disease stage, smoking status, body mass index, age at first birth and parity, oral contraceptive use, menopausal status and use of hormone replacement, radiation treatment, systemic treatment with chemotherapy and/or hormonal therapy	8
Holmes et al. (2013b)	Canada	ACEI, ARB, BB, CCB, Diuretics vs. non	2004–2010	Provincial Cancer Registry data	4019	Retrospective cohort study	BCSM	Age, stage at diagnosis, history of previous cancer, and urban/rural residence	7
Melhem-Bertrandt et al. (2011)	United States	ACEI, ARB, BB vs. non	1995–2007	The Breast Cancer Management System Database	1413	Cohort study	OS,RFS	Age, race, stage, grade, receptor status, lymphovascular invasion, body mass index, diabetes, hypertension, and angiotensin-converting enzyme inhibitor use	7
Modiet al. (2020)	Australia	BB vs. non	–	Clinical trials EMILIA, TH3RESA, MARIANNE, and CLEOPATRA	2777	Retrospective study	OS	Age, Race, BMI, Albumin count, ECOG PS, ER/PR Status, Presence of Visceral Disease and Brain Metastasis, Arrhythmia, Coronary Artery Disease, Heart Failure, Hypertension, Cerebrovascular disease, Diabetes and Other Cardiovascular diseases, Prior treatment to anthracyclines, taxanes and trastuzumab	8
Musselman et al. (2018)	Canada	BB vs. non	2002–2010	The Institute for Clinical and Evaluative Sciences	4876	Cohort study	BCSM	Statin use and socioeconomic status	7
Powe et al. (2010)	United Kingdom	BB vs. non	1987–1994	The Nottingham City Hospital	466	Cohort study	DSS, recurrence	Adjuvant therapy and age	6
Sakellakis et al. (2014)	Greece	BB vs. non	1983–2013	Department of Medicine, University Hospital Patras Medical School	610	Retrospective study	DFS	Age, tumor stage, hormone receptor status and HER2 status	6
Şendur et al. (2012)	Turkey	ARB vs. non	2004–2011	Department of Medical Oncology, Ankara Numune Education and Research Hospital	486	Cohort study	OS, DFS	NA	7
Shah et al. (2011)	United Kingdom	BB vs. other AHT	1997–2007	The Doctors Independent Network Database	3462	Cohort study	OS	Age, gender, year of diagnosis, smoking status, number of medications received in the year before diagnosis, area deprivation and national region	7
Shih et al. (2020)	China	CCB vs. non	2007–2015	The National Health Insurance Research Database	4840	Case-control	recurrence	Demographic characteristics, comorbidities, and tumor-node-metastasis stage, age, monthly income, geographic region, urbanization level, hypertension, hyperlipidemia, diabetes, chronic kidney disease, tumor-node-metastasis (TNM) classification of malignant tumors, and index date	7
Santala et al. (2020)	Finland	ACEI, ARB, BB, CCB, Diuretics vs. non	1995–2013	Finnish Cancer Registry	73,170	Cohort study	BCSM	Age at diagnosis, tumor extent, charlson-comorbidity index, primary treatment of breast cancer, obesity, participation in national screening program and use of hormone-receptor antagonists after breast cancer diagnosis	7
Sorensen et al. (2013)	Denmark	ACEI, ARB, BB vs. non	1996–2003	The Danish Breast Cancer Cooperative Group registry	18,733	Cohort study	recurrence	Age at diagnosis, menopausal status at diagnosis, UICC stage, histologic grade, ER status and receipt of adjuvant endocrine therapy, receipt of adjuvant chemotherapy, type of primary surgery received, Charlson comorbidity index, prediagnosis combination HRT, and coprescriptions of any BB, ACEI, ARB, aspirin, and simvastatin	8
Spera et al. (2017)	Canada	BB vs. non	NA	The ROSE/TRIO-012 study, BCIRG-005 data	1144	Retrospective study	PFS, OS	Age, Eastern Cooperative Oncology Group performance status and menopausal status	7
Springate et al. (2015)	United Kingdom	BB vs. other AHT	1997–2006	The Clinical Practice Research Datalink (CPRD) and Doctors’ Independent Network (DIN)	14,964	Cohort study	BCSM	Patient age, gender, year of diagnosis, smoking status, number of medications received in year prior to diagnosis, Regional Health Authority, and practice postcode Index of Multiple Deprivation, cancer site prevalence, year of diagnosis and area deprivation	7
Sorensen et al. (2000)	Denmark	CCB vs. non	1989–1995	The Danish Cancer Registry, and the Danish Death Registry	23,167	Cohort study	Risk, BCSM	NA	6
Takada et al. (2019)	Japan	CCB, RASI vs. non	2007–2018	The Osaka City University Hospital	338	Retrospective study	DFS, OS	NA	7
Wei et al. (2020)	United States	Diuretics vs. non	2005–2017	Humana Insurance database	1492	Retrospective study	recurrence	Age, spironolactone, alopecia, acne vulgaris, hirsutism, hypertension, congestive heart failure, primary aldosteronism, nephrotic syndrome, ascites, alcohol abuse, smoking, illicit drug abuse, and insurance plan	7

Abbreviations: NOS, Newcastle-Ottawa Scale; ACEI, angiotensin-converting enzyme inhibitors; ARB, angiotensin II receptor blockers; CCB, calcium-channel blockers; BB, beta-blockers; RASI, renin-angiotensin system inhibitors; BCSM, breast cancer-specific mortality; OS, overall survival; DSS, disease-specific survival; DFS, disease-free survival; NA, Not Available.

### Association of Antihypertensive Medication Use with Breast Cancer Risk

**FIGURE 2:**
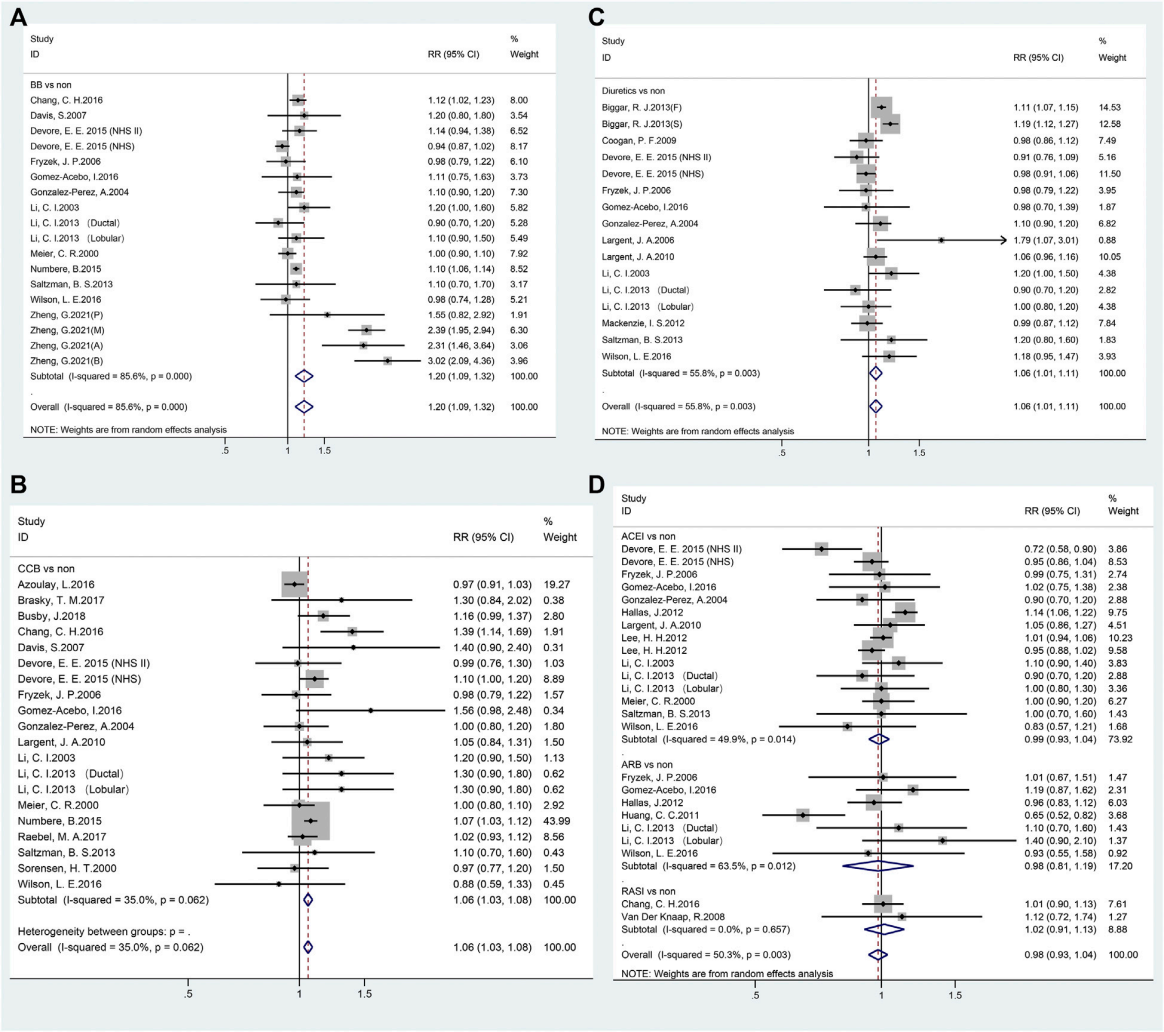
Forest plot of studies among the risk of breast cancer with antihypertensive medications use. Legends (**A** beta-blockers; **B**: calcium-channel blockers; **C**: diuretics; **D**: renin-angiotensin system inhibitors).

Pooled data from 18 studies related to breast cancer risk showed that the use of BBs was associated with increased cancer risk (RR = 1.20, 95% CI 1.09–1.32; Figure 2A and Table 2). A subgroup analysis showed a significant association with case-control (RR = 1.09, 95% CI 1.05–1.12), nested case-control (RR = 1.11, 95% CI 1.03–1.20) studies, and cohort (RR = 1.43, 95% CI 1.07–1.91) studies. In addition, a significant association was observed in Europe (RR = 1.41, 95% CI 1.16–1.70) and Asia (RR = 1.12, 95% CI 1.02–1.23), but not in North America (RR = 1.02, 95% CI 0.94–1.11). However, subgroup analysis of the duration of administration in years showed that the use of BBs even for ≥10 years was not associated with breast cancer risk.

**TABLE 2:** The results of the association between antihypertensive medication use and breast cancer risk.

Comparison	BB vs. non				CCB vs. non				Diuretics vs. non				RASI vs. non			
Category	N	RR (95%CI)	*I* ^*2*^ (%)	*P*	N	RR (95%CI)	*I* ^*2*^ (%)	*P*	N	RR (95%CI)	*I* ^*2*^ (%)	*P*	N	RR (95%CI)	*I* ^*2*^ (%)	*P*
Risk	19	**1.20 (1.09–1.32)**	32.4	0.116	20	**1.06 (1.03–1.08)**	35.0	0.062	16	**1.06 (1.01–1.11)**	55.8	0.003	24	0.98 (0.93–1.04)	50.3	0.003
**Study design**																
Case-control study	7	**1.09 (1.05–1.12)**	0.0	0.455	9	**1.09 (1.05–1.13)**	7.7	0.371	6	1.04 (0.92–1.18)	39.3	0.144	12	1.03 (0.97–1.09)	40.0	0.074
Cohort study	9	**1.43 (1.07–1.91)**	0.0	0.457	9	1.01 (0.97–1.05)	0.0	0.648	9	1.06 (1.00–1.13)	66.7	0.002	10	0.89 (0.80–1.01)	48.6	0.041
Nest case-control study	2	**1.11 (1.03–1.20)**	0.0	0.837	2	**1.18 (1.03–1.36)**	80.8	0.022	1	1.10 (0.95–1.27)	–	–	2	0.99 (0.89–1.10)	0.0	0.440
**Geographic area**																
North America	8	1.02 (0.94–1.11)	17.5	0.292	11	**1.08 (1.02–1.14)**	0.0	0.661	10	1.03 (0.97–1.10)	35.8	0.122	11	0.96 (0.88–1.05)	24.9	0.206
Europe	9	**1.41 (1.16–1.70)**	0.0	0.434	8	**1.04 (1.01–1.07)**	45.3	0.077	6	**1.10 (1.04–1.17)**	47.5	0.090	9	1.07 (1.00–1.13)	5.8	0.387
Asia	1	**1.12 (1.02–1.23)**	–	–	1	**1.39 (1.14–1.69)**	–	–					4	0.93 (0.84–1.04)	79.1	0.002
**Duration of use**																
<5 years	9	1.05 (0.99–1.11)	0.0	0.49	11	1.04 (0.93–1.16)	50.7	0.027	9	1.04 (0.98–1.11)	0.0	0.901	14	**0.94 (0.90–0.99)**	0.0	0.736
5–10 years	10	0.98 (0.89–1.07)	0.0	0.908	12	1.09 (0.99–1.19)	0.0	0.854	10	1.03 (0.90–1.19)	58.1	0.011	18	0.98 (0.86–1.11)	67.0	0.000
≥10 years	7	1.08 (0.91–1.29)	59.0	0.023	8	1.05 (0.72–1.53)	62.9	0.009	7	**1.10 (1.01–1.20)**	0.0	0.652	10	**0.78 (0.68–0.91)**	0.0	0.563

Abbreviations: BB, beta-blockers; CCB, calcium-channel blockers; RASI, renin-angiotensin system inhibitors; RR, relative risk; CI, confidence intervals; N, number of study.

Twenty studies reported the relationship between CCBs and breast cancer risk (Figure 2B; Table 2). The pooled RR of breast cancer risk for CCBs use vs. non-use was 1.06 (95% CI 1.03–1.08), indicating a significant positive association. Case-control studies (RR = 1.09, 95% CI 1.05–1.13), nested case-control studies (RR = 1.18, 95% CI 1.03–1.36), and every area (North America: RR = 1.08, 95% CI 1.02–1.14; Europe: RR = 1.04, 95% CI 1.01–1.07; Asia: RR = 1.39, 95% CI 1.14–1.69) showed a positive association. There was no duration-response association between long-term use of CCB and breast cancer risk.

As shown in Figure 2C, exposure to diuretics (16 studies) showed a significant association where the pooled RR was 1.06 (95% CI 1.01–1.11). A significant relationship was noted in Europe (RR = 1.10, 95% CI 1.04–1.17) but not in North America (RR = 1.03, 95% CI 0.97–1.10). Notably, a significant association was observed with long-term use of diuretics (≥10 years; RR = 1.10, 95% CI 1.01–1.20, Table 2).

A total of 24 studies reported a connection between RASIs exposure of any duration and breast cancer risk. RASIs did not increase the risk of breast cancer with a pooled RR (0.98, 95% CI 0.93–1.04), as shown in Figure 2D. However, it is worth noting that RASIs played a protective role in breast cancer when studies were restricted to those where the duration of RASI use was <5 years (RR = 0.94, 95% CI 0.90–0.99) and ≥10 years (RR = 0.78, 95% CI 0.68–0.91). Fifteen and seven studies were related to ACEI and ARB use, respectively, and the results suggested that use of ACEI or ARB was not related to breast cancer risk (ACEI: RR = 0.99, 95% CI 0.93–1.04; ARB: RR = 0.98, 95% CI 0.81–1.19, Table 2).

### Association of Antihypertensive Medication Use with Breast Cancer Prognosis Breast Cancer-Specific Mortality

**FIGURE 3:**
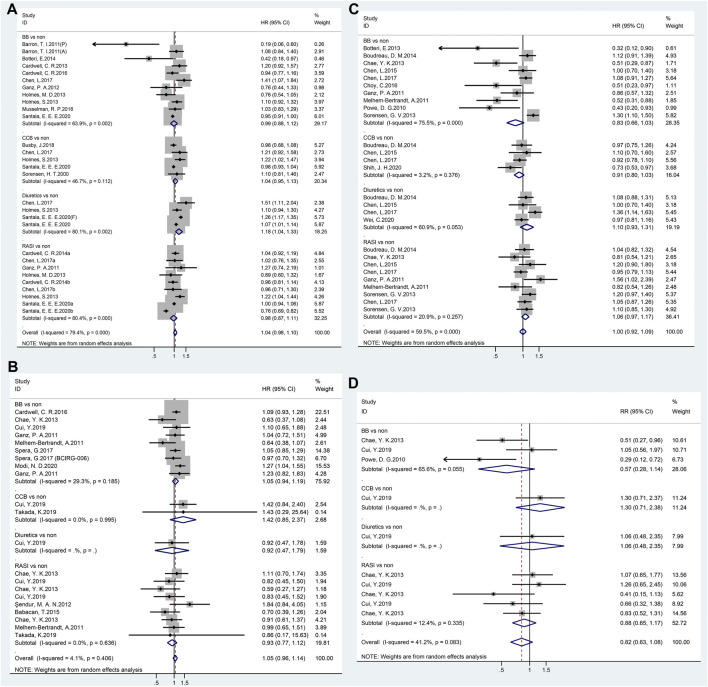
Forest plot of studies among the prognosis of breast cancer patients with antihypertensive medications use. Legends (**A**: breast cancer-specific mortality; **B**: overall survival; **C**: recurrence; **D**: disease-specific survival).

Twenty-nine independent studies examined the association between antihypertensive medication use and breast cancer-specific mortality, with a pooled HR of 1.04 (95% CI 0.98–1.10). When HRs for the individual antihypertensive medication (BBs, CCBs, and RASIs) were calculated in subgroup analyses, no significant results were observed (Figure 3A). However, a significant association was observed in diuretic users (HR = 1.18, 95% CI 1.04–1.33).

### OS

Twenty-one studies revealed no significant link between antihypertensive medication use and breast cancer OS (HR = 1.05, 95% CI 0.96–1.14, Figure 3B). Furthermore, a subgroup analysis of the classes of antihypertensive medication showed no significant association was observed.

### Recurrence

In total, 27 studies reported breast cancer recurrence with the use of antihypertensive medication. However, as shown in Figure 3C, no effect was observed for any type of antihypertensive medication (HR = 1.00, 95% CI 0.92–1.09).

### DSS

The results of the meta-analyses on DSS are summarized in forest plots for all 10 studies; no association was found between antihypertensive medication use and breast cancer DSS (HR = 0.82, 95% CI 0.63–1.08, Figure 3D).

### Risk of Publication Bias

The results of the Begg’s funnel plot and Egger’s regression test, showed no evidence of potential publication bias in articles reporting the use of BBs (*P* = 0.168), CCBs (*P* = 0.151), diuretics (*P* = 0.348), or RASIs (*P* = 0.397) in relation to the risk of breast cancer risk (Supplementary Figure S1). In addition, a potential publication bias was observed in studies on the use of antihypertensive medication with recurrence (*P* = 0.001), but not in other studies on prognosis (mortality: *P* = 0.717; OS: *P* = 0.096; DSS: *P* = 0.125) (Supplementary Figure S2).

### Sensitivity Analysis

A sensitivity analysis was performed as shown in Supplementary Figures S3 and S4. There was no significant alteration in the pooled RRs or HRs after sequentially deleting a single study from the overall pooled analyses each time. In the sensitive analysis of diuretics users with risk of breast cancer, the pooled RR was not statistically significant after the deletion of an article (RR = 1.03, 95% CI 0.98–1.08) (Biggar et al., 2013).

## Discussion

Our meta-analysis showed that the use of BBs, CCBs, or diuretics was associated with a moderate increase in the risk of breast cancer. Diuretic therapy for ≥10 years could increase breast cancer risk by 10%. Long-term use of RASIs exerted a protective effect against breast cancer, especially when used for ≥10 years. In addition, this meta-analysis indicated that diuretics are related to elevated breast cancer-specific mortality, but antihypertensive medication use did not affect recurrence, OS, or DSS.

Our conclusion that BBs were associated with increased breast cancer risk was consistent with this highly influential and large studies (Numbere et al., 2015; Chang et al., 2016; Zheng et al., 2021). The prospective study data minimizes the recall bias and in the study in question, some important confounders such as alcohol status, smoking status, BMI, medication and comorbidities, were adjusted. Similarly, the positive association between diuretics and breast cancer risk mainly depended on the study that evaluated cancer risk in 2.3 million Danish women who were followed-up for 28.8 million person-years (Biggar et al., 2013). The study reported that breast cancer risk increased in the first-year of diuretic use, but the association was attributed to reverse causality, such as where symptoms of cancer, including abdominal swelling or incidental findings such as hypertension, led to diuretic exposure. To exclude care practices that affect the risk from true etiologic impact, the study further examined risk only in women who were exposed to drugs for more than one year prior to diagnosis, and found a slightly increased risk of breast cancer with increasing duration of diuretic exposure. Consistent with its results, we observed a duration-response association between the duration of diuretic therapy ≥10 years and breast cancer risk. Studies found that thiazide diuretics, but not other diuretics, were linked to an increasing risk of breast cancer, and the authors considered the increase in insulin resistance due to thiazide diuretics as a possible explanation (Sarafidis et al., 2007; Ni et al., 2017). A recent study showed that the use of furosemide or other diuretics before breast cancer diagnosis increased the risk of breast cancer-related death (Santala et al., 2020). Our results showed that the breast cancer mortality increased with the use of diuretics; however, we failed to analyze the effect of diuretic subtypes on breast cancer prognosis because there were few relevant studies. Thus, we could not arrive at the conclusion that different subclass of diuretics had varying associations with breast cancer. Future studies are warranted to determine the potential difference among various diuretics.

Our meta-analysis showed a positive association between CCBs use and the risk of breast cancer, which was consistent with the results of previous studies (Wright et al., 2017; Busby et al., 2018a). The elevated levels of cytosolic calcium could affect the process of apoptosis through different signaling pathways such as caspase activation, induction of endonuclease activity, or the miRNA-524-5p-BRI3-extracellular signal-regulated kinase (Erk) pathway (Iwasawa et al., 2011; Guo et al., 2014; Zhao et al., 2017b). CCBs blocks calcium entry, inhibiting this vital process, which could destroy the body’s natural defense mechanism against cancer growth and promote cell survival by initiating autophagy (Sun et al., 2018). However, there is no conclusive evidence to accurately explain this observed link and the benefits of the long-term use of CCBs are controversial.

The association between RASIs and breast cancer remains controversial with some studies reporting that ARB/ACEI was not related to breast cancer risk (Teo, 2011; Datzmann et al., 2019), whereas another concluded that users of ACEIs had an increased risk (Hallas et al., 2012). However, our result demonstrated a significant (22%) reduction in breast cancer risk with ≥10 years, which is consistent with previously published observational studies (Huang et al., 2011; Li et al., 2013; Wang et al., 2013; Ni et al., 2017). In addition, *in vivo* and *in vitro* studies on RASIs have demonstrated a protective anti-inflammatory, anti-proliferative, and pro-apoptotic effect on breast cancer (Herr et al., 2008; George et al., 2010). Although the effect of RASIs on breast cancer is still contradictory, RASIs should be selected more often for patients undergoing antihypertensive therapy considering its availability, feasibility, and established safety with reduced breast cancer risk.

It is important to assess the possible source of heterogeneity. First, the significance of our results might have been affected by an indication bias, such as high blood pressure (Coogan, 2013), as both hypertension and cancer are associated with obesity, alcohol, smoking, cardiometabolic abnormalities, and other comorbidities. One study reported a relationship between hypertension and cancer (Han et al., 2017). In addition, in this meta-analysis, not all included studies adjusted for the confounding factor of hypertension which would cause a certain degree of heterogeneity. Second, in observational studies of the general population, it is important to determine a dose or duration-response relationship to properly interpret the potential link between drug exposure and cancer development. However, because of the differences in drug exposure time and dose between the included studies, the results will eventually be heterogeneous. Third, most of studies were from North America or Europe but a few studies are from Asia, which produced regional heterogeneity and limited the extent to which the results can be generalized. In the subgroup analysis according to geographical area, we found reduced between-study heterogeneity and observed different results, which indicated that the association varied among different areas. Finally, because the included studies were retrieved from some population registration database, the analysis results could reduce recall bias and misclassification bias to some extent. However, population-based registries also have some shortcomings, such as the limitation of potential confounders by stored data, and the fact that a prescription was collected does not necessarily indicate that the prescribed drugs were taken.

Our meta-analysis provides convincing and clear evidence of the relationship between antihypertensive drugs and the risk and prognosis of breast cancer. One strength of our study is that we conducted a stratified analysis according to the duration of antihypertensive drug use and found that using diuretics for ≥10 years could increase the risk of breast cancer, but using RASIs for ≥10 years would reduce the risk of breast cancer. This suggests that RASIs would be the best choice for patients with long-term antihypertensive drugs. Another strength of the study is that we focused on breast cancer risk and prognosis to obtain a more homogeneous group of studies. We studied the possible source of heterogeneity between studies, including study design, geographic area, and duration of antihypertensive medication use. It is noteworthy that there were also some limitations to this study. First, biases such as confounders, possible detection bias, and selection bias is inevitable in observational studies. Secondly, subgroup analyses based on age, sex, pathology of breast cancer, dose of antihypertensive medication, subclass of BB (selective BBs and non-selective BBs), CCBs (dihydropyridines and non-dihydropyridines), and diuretics (thiazide, loop, and potassium sparing diuretics) were not conducted because of the lack of relevant studies. Thirdly, our current summary evidence did not support a clear relation between long-term use of CCBs or BBs and breast cancer risk. Finally, significant heterogeneity was observed among studies focused on antihypertensive medication use and breast cancer risk and this persisted even when the data were stratified into subgroups.

The results of this meta-analysis demonstrated that BBs, CCBs, and diuretics were significantly associated with breast cancer risk. Unexpectedly, a beneficial effect of long-term use of RASI was observed against breast cancer risk. Additionally, according to the analysis of prognosis, no association was observed with any class of antihypertensive medication. Considering the limitations in this meta-analysis, further research is needed to fully clarify the association between antihypertensive medication use and breast cancer risk.

## Data Availability

The raw data supporting the conclusions of this article will be made available by the authors, without undue reservation.
