# Supplementary material for: Association Between Antihypertensive Medication Use and Breast Cancer: A Systematic Review and Meta-Analysis
Source: Front Pharmacol. 2021 May 13;12:609901. doi: 10.3389/fphar.2021.609901 (PMC8155668; doi:10.3389/fphar.2021.609901)
Supplement: Supplementary file 1 [file Table3.doc]

**Supplementary table 3.** Quality assessment of eligible studies by Newcastle-Ottawa Scale (NOS).

| **Author, Year** | **Design Type** | **Selection** | **Comparability** | **Exposure** | **Total NOS** |
| --- | --- | --- | --- | --- | --- |
| Brasky et al, 2017 | case-control | 3 | 2 | 2 | 7 |
| Barron et al, 2011 | case-control | 4 | 1 | 3 | 8 |
| Busby et al, 2018 | case-control | 3 | 2 | 2 | 7 |
| Chae et al, 2013 | case-control | 3 | 1 | 3 | 7 |
| Coogan et al, 2009 | case-control | 3 | 2 | 3 | 8 |
| Davi et al, 2007 | case-control | 2 | 1 | 3 | 6 |
| Gomez-Acebo et al, 2016 | case-control | 2 | 2 | 3 | 7 |
| Hallas et al, 2012 | case-control | 4 | 1 | 3 | 8 |
| Largent et al, 2006 | case-control | 3 | 1 | 2 | 6 |
| Lee et al, 2012 | case-control | 3 | 1 | 3 | 7 |
| Li et al, 2003 | case-control | 3 | 1 | 3 | 7 |
| Li et al, 2013 | case-control | 4 | 1 | 3 | 8 |
| Meier et al, 2000 | case-control | 3 | 2 | 2 | 7 |
| Numbere et al, 2015 | case-control | 4 | 1 | 2 | 7 |
| Shih et al, 2020 | case-control | 2 | 2 | 3 | 7 |
| **Author, Year** | **Design Type** | **Selection** | **Comparability** | **Outcome** | **Total NOS** |
| Azoulay et al, 2016 | cohort study | 3 | 2 | 2 | 7 |
| Babacan et al, 2015 | cohort study | 3 | 1 | 2 | 7 |
| Biggar et al, 2013 | cohort study | 3 | 1 | 2 | 6 |
| Botteri et al, 2013 | cohort study | 2 | 2 | 3 | 7 |
| Boudreau et al, 2014 | cohort study | 3 | 2 | 3 | 8 |
| Busby et al, 2018 | cohort study | 3 | 2 | 2 | 7 |
| Cardwell et al, 2016 | cohort study | 4 | 1 | 2 | 7 |
| Chen et al, 2017 | cohort study | 3 | 2 | 2 | 7 |
| Choy et al, 2016 | cohort study | 3 | 1 | 2 | 6 |
| Cui et al, 2019 | cohort study | 3 | 2 | 2 | 7 |
| Devore et al, 2015 | cohort study | 3 | 2 | 3 | 8 |
| Fryzek et al, 2006 | cohort study | 3 | 1 | 3 | 7 |
| Ganz et al, 2011 | cohort study | 2 | 2 | 3 | 7 |
| Holmes et al, 2013 | cohort study | 3 | 2 | 3 | 8 |
| Holmes et al, 2013 | cohort study | 3 | 1 | 3 | 7 |
| Huang et al, 2011 | cohort study | 3 | 2 | 2 | 7 |
| Largent et al, 2010 | cohort study |  |  |  | 7 |
| Mackenzie et al, 2012 | cohort study | 3 | 2 | 2 | 7 |
| Melhem-Bertrandt et al, 2011 | cohort study | 3 | 1 | 3 | 7 |
| Modiet al, 2020 | cohort study | 3 | 2 | 3 | 8 |
| Musselman et al, 2018 | cohort study | 3 | 1 | 3 | 7 |
| Powe et al, 2010 | cohort study | 2 | 1 | 3 | 6 |
| Raebel et al, 2017 | cohort study | 3 | 1 | 2 | 6 |
| Sakellakis et al, 2014 | cohort study | 3 | 1 | 2 | 6 |
| Saltzman et al, 2013 | cohort study | 3 | 1 | 3 | 7 |
| Santala et al, 2020 | cohort study | 3 | 2 | 2 | 7 |
| Şendur et al, 2012 | cohort study | 3 | 1 | 3 | 7 |
| Shah et al, 2011 | cohort study | 4 | 1 | 2 | 7 |
| Sorensen et al, 2000 | cohort study | 3 | 1 | 2 | 6 |
| Sorensen et al, 2013 | cohort study | 3 | 2 | 3 | 8 |
| Spera et al, 2017 | cohort study | 3 | 1 | 3 | 7 |
| Springate et al, 2015 | cohort study | 3 | 1 | 3 | 7 |
| Takada et al, 2019 | cohort study | 3 | 1 | 3 | 7 |
| Van Der Knaap et al, 2008 | cohort study | 3 | 2 | 2 | 7 |
| Wei et al, 2020 | cohort study | 3 | 1 | 3 | 7 |
| Wilson et al, 2016 | cohort study | 3 | 2 | 3 | 8 |
| Zheng, G et al, 2021 | cohort study | 3 | 1 | 3 | 7 |
| Cardwell et al, 2013 | nested case-control | 3 | 2 | 3 | 8 |
| Cardwell et al, 2014 | nested case-control | 3 | 2 | 3 | 8 |
| Chang et al, 2016 | nested case-control | 3 | 2 | 3 | 8 |
| Chen et al, 2015 | nested case-control | 3 | 2 | 2 | 7 |
| Gonzalez-Perez et al, 2004 | nested case-control | 4 | 2 | 2 | 8 |
