# Supplementary material for: Association Between Antihypertensive Medication Use and Breast Cancer: A Systematic Review and Meta-Analysis
Source: Front Pharmacol. 2021 May 13;12:609901. doi: 10.3389/fphar.2021.609901 (PMC8155668; doi:10.3389/fphar.2021.609901)

**Supplementary Figure 1.** Begger’s funnel plot of risk studies included in this meta-analysis. Legends: (A: beta-blockers; B: calcium-channel blockers; C: diuretics; D: renin-angiotensin system inhibitors)


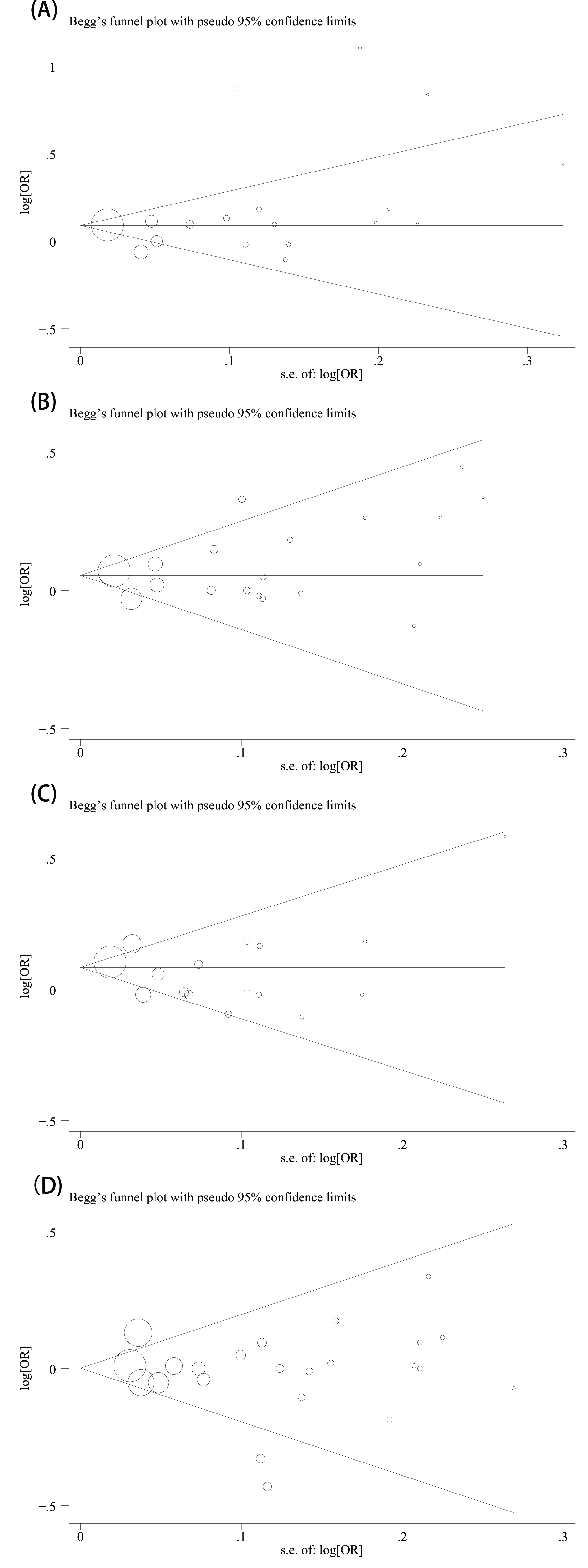


**Supplementary Figure 2.** Begger’s funnel plot of prognosis studies included in this meta-analysis. Legends: (A: breast cancer-specific mortality; B: recurrence; C: overall survival; D: disease-specific survival)


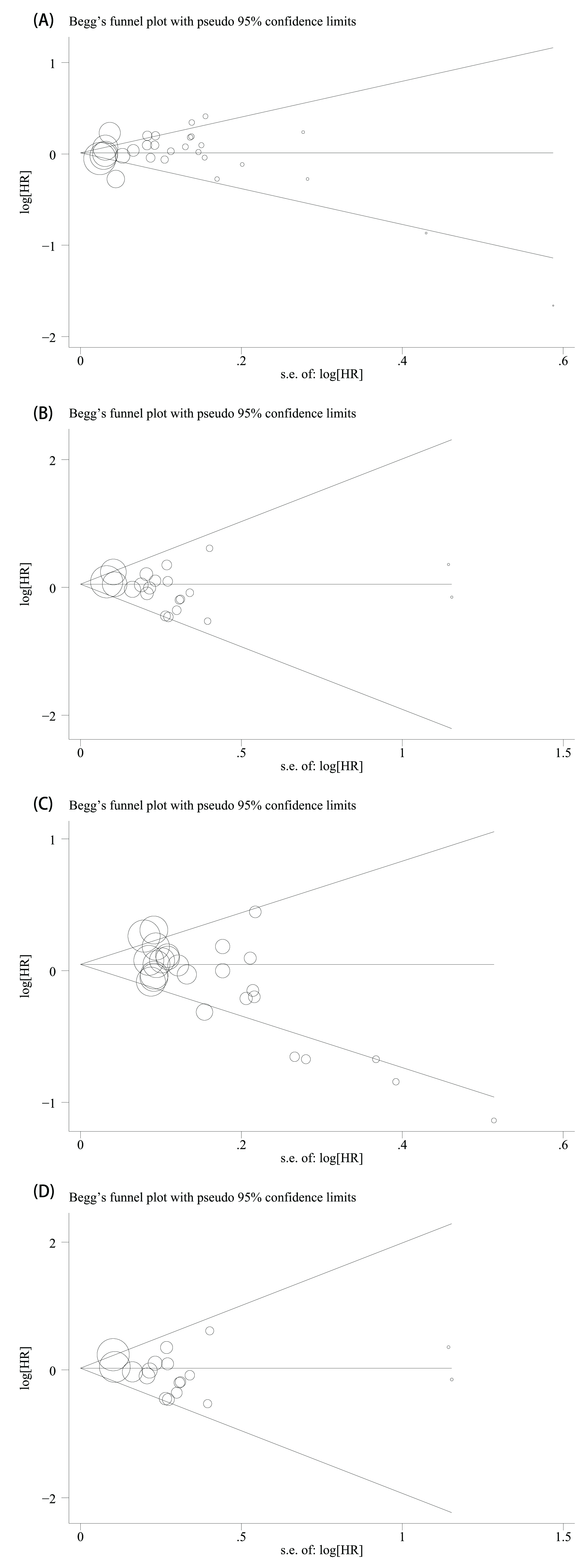


**Supplementary Figure 3.** Sensitivity analysis of the effect of the individual study on the risk studies. Legends: (A: beta-blockers; B: calcium-channel blockers; C: diuretics; D: renin-angiotensin system inhibitors)


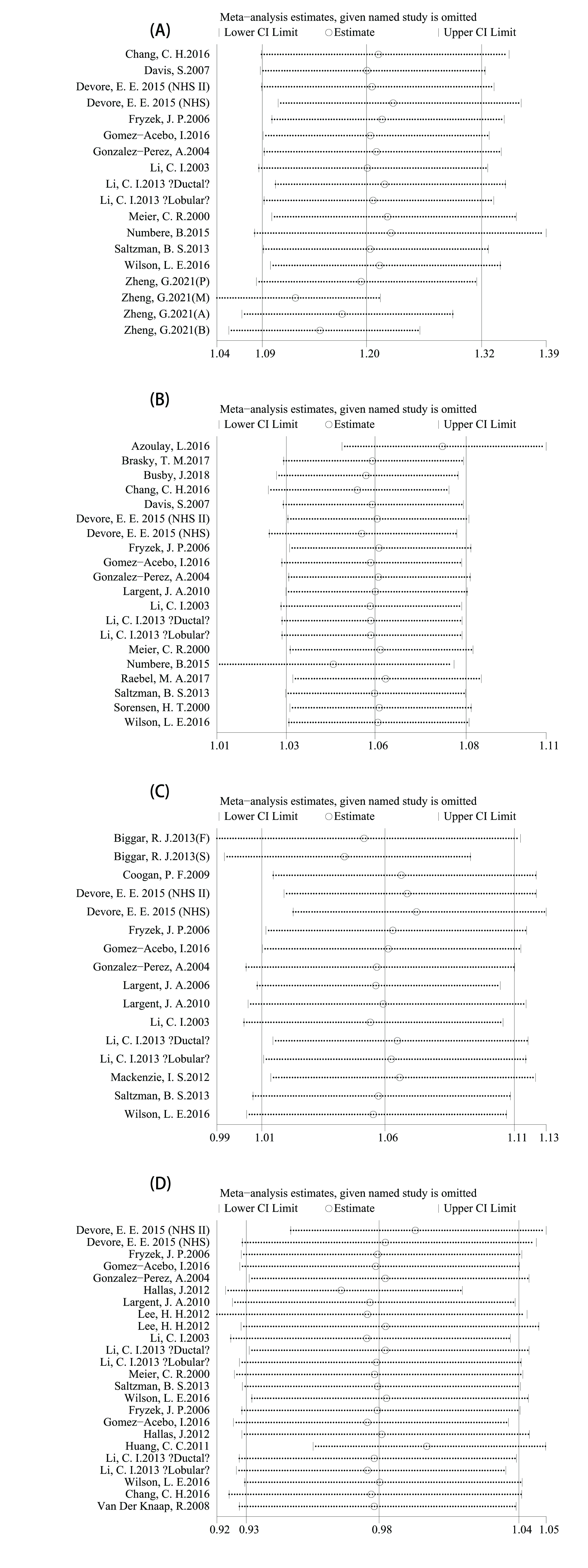


**Supplementary Figure 4.** Sensitivity analysis of the effect of the individual study on the prognosis studies. Legends: (A: breast cancer-specific mortality; B: recurrence; C: overall survival; D: disease-specific survival)


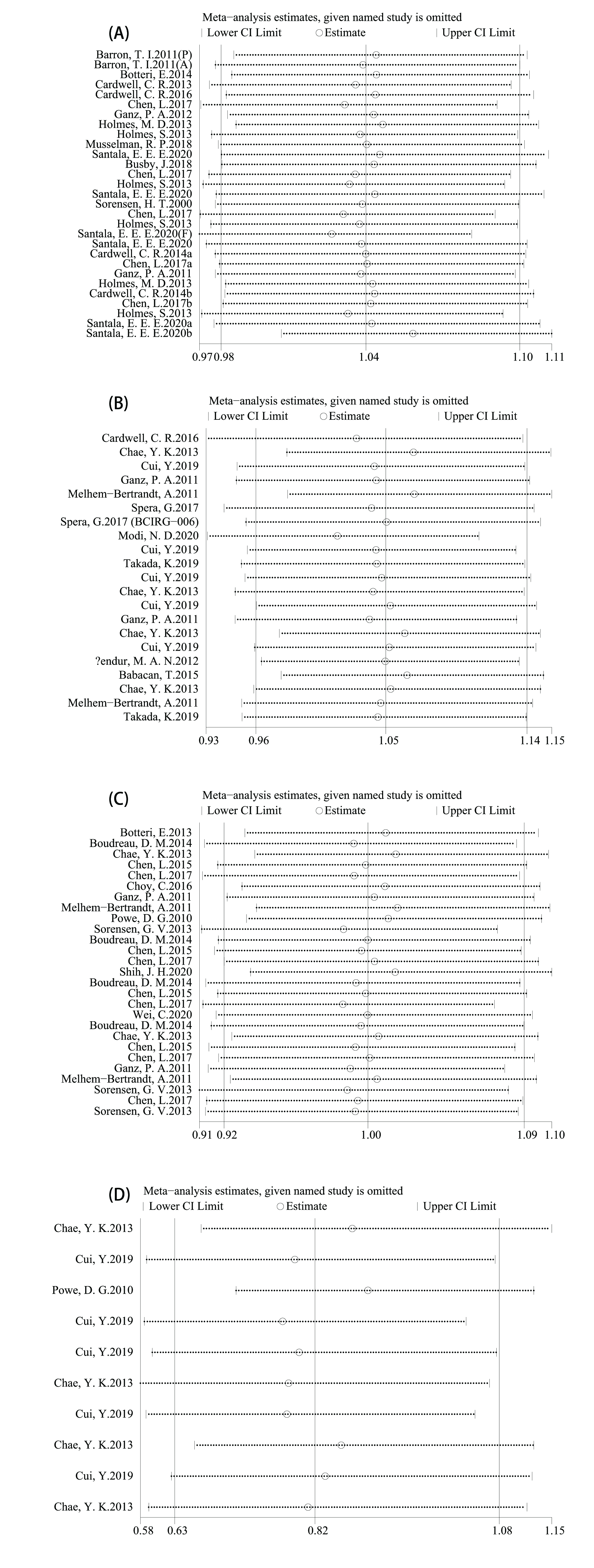

Supplement: Supplementary file 2 [file DataSheet1.doc]
