# Supplementary material for: Association Between Antihypertensive Medication Use and Breast Cancer: A Systematic Review and Meta-Analysis
Source: Front Pharmacol. 2021 May 13;12:609901. doi: 10.3389/fphar.2021.609901 (PMC8155668; doi:10.3389/fphar.2021.609901)
Supplement: Supplementary file 3 [file Table1.doc]

**Supplementary table 1: search strategy**

| **#1** | “Cancer” or “Carcinoma” or “Malignancy” or “Neoplasm” or “Tumor” |
| --- | --- |
| **#2** | “Breast” |
| **#3** | **#1 AND #2** |
| **#4** | “Antihypertensive Drug” or “Antihypertensive Agent” or “Antihypertensive Medication” |
| **#5** | “ARBs” or “ARB” or “Angiotensin Receptor Antagonists” or “Angiotensin Receptor Blocker” or “Angiotensin-Receptor Blocker” or “Angiotensin Receptor Blockers” or “Angiotensin Receptor Antagonist” or “Angiotensin II Receptor Blocker” or “Angiotensin II Receptor Blockers” or “Angiotensin Receptor Blockade” or “Renin Angiotensin System Inhibitor” or “Renin-Angiotensin System Inhibitor” or “RAS Inhibitor” or “Candesartan” or “Eprosartan” or “Irbesartan” or “Losartan” or “Olmesartan” or “Tasosartan” or “Telmisartan” or “Valsartan” |
| **#6** | “ACEI” or “ACEIs” or “Angiotensin-Converting Enzyme Inhibitors” or “Angiotensin Converting Enzyme Inhibitor” or “ACE Inhibitor” or “Benazepril” or “Captopril” or “Delapril” or “Ramipril” or “Cilazapril” or “Enalapril” or “Fosinopril” or “Perindopril” or “Imidapril” or “Lisinopril” or “Moexipril” or “Quinapril” or “Trandolapril” or “Spirapril” or “Temocapril” or “Zofenopril” |
| **#7** | “Beta Blockers” or “Beta-Blocker” or “Beta-Receptor Antagonist” or “Beta Adrenergic Antagonists” or “Acebutolol” or “Atenolol” or “Bisoprolol” or “Carvedilol” or “Celiprolol” or “Esmolol” or “Labetalol” or “Metoprolol” or “Nadolol” or “Nebivalol” or “Propanolol” or “Sotalol” or “Timolol” |
| **#8** | “Calcium Channel Blockers” or “Calcium Channel Blocker” or “Calcium Channel Antagonists” or “Calcium Channel Antagonist” or “Amlodipine” or “Benidipine” or “Diltiazem” or “Felodipine” or “Isradipine” or “Manidipine” or “Nicardipine” or “Nifedipine” or “Nisoldipine” or “Nitrendipine” or “Verapamil” |
| **#9** | “Diuretics” or “Chlorothiazide” or “Hydrochlorothiazide” or “Bendroflumethiazide” or “Hydroflumethiazide” or “Methylchlothiazide” or “Polythiazide” or “Trichlormethiazide” or “Chlorthalidone” or “Metolazone” or “Indapamide” |
| **#10** | “RASI” or “RASIs” or “renin-angiotensin system inhibitors” or “renin angiotensin system inhibitors” or “RAS inhibitor” |
| **#11** | #4 OR #5 OR #6 OR #7 OR #8 OR #9 OR #10 |
| **#12** | “incidence” or “risk” or “prognoses” or “prognosis” or “prognostic” or “survival” or “mortality” or “recurrence” or “overall survival” or “disease specific survival” |
| **#13** | **#3 AND #11 AND #12** |

The search strategy was adapted for each electronic database.
